# *Drosophila* tubulin polymerization promoting protein mutants reveal pathological correlates relevant to human Parkinson’s disease

**DOI:** 10.1038/s41598-021-92738-3

**Published:** 2021-06-30

**Authors:** Jing Xie, Shuting Chen, Jean C. Bopassa, Swati Banerjee

**Affiliations:** 1 Department of Cellular and Integrative Physiology, Joe R. and Teresa Lozano Long School of Medicine, University of Texas Health Science Center San Antonio, 7703 Floyd Curl Drive, San Antonio, TX 78229 USA; 2 Xiangya School of Medicine, Central South University, Changsha, 410083 Hunan China

**Keywords:** Cell biology, Neuroscience

## Abstract

Parkinson’s disease (PD) is a progressive neurodegenerative disorder with no known cure. PD is characterized by locomotion deficits, nigrostriatal dopaminergic neuronal loss, mitochondrial dysfunctions and formation of α-Synuclein aggregates. A well-conserved and less understood family of Tubulin Polymerization Promoting Proteins (TPPP) is also implicated in PD and related disorders, where TPPP exists in pathological aggregates in neurons in patient brains. However, there are no in vivo studies on mammalian TPPP to understand the genetics and neuropathology linking TPPP aggregation or neurotoxicity to PD. Recently, we discovered the only *Drosophila* homolog of human TPPP named Ringmaker (Ringer). Here, we report that adult *ringer* mutants display progressive locomotor disabilities, reduced lifespan and neurodegeneration. Importantly, our findings reveal that Ringer is associated with mitochondria and *ringer* mutants have mitochondrial structural damage and dysfunctions. Adult *ringer* mutants also display progressive loss of dopaminergic neurons. Together, these phenotypes of *ringer* mutants recapitulate some of the salient features of human PD patients, thus allowing us to utilize *ringer* mutants as a fly model relevant to PD, and further explore its genetic and molecular underpinnings to gain insights into the role of human TPPP in PD.

## Introduction

Parkinson’s disease (PD) is a debilitating neurodegenerative disorder. The limited availability of animal models recreating the progressive pathology of PD have hindered the development of effective disease-modifying therapies. Thus, there is a significant unmet need for experimental paradigms that will allow for the identification of mediators/regulators of genes and mechanisms that underlie PD. Among the well-defined characteristics of PD are locomotion deficits, loss of dopaminergic neurons in substantia nigra, mitochondrial dysfunctions^1,2^ and aggregation of α-Synuclein (α-Syn) in neuronal inclusions in patient brains^3,4^. Interestingly, a highly conserved family of human Tubulin Polymerization Promoting Proteins (TPPP) is also reported to localize in pathological aggregates in PD^3,5,6^.

TPPPs are a superfamily of microtubule-associated proteins with a common C-terminus p25α domain^7^. TPPP interacts with Tubulin and promotes bundling and stabilization of microtubules in vitro and in vivo^8–10^. Human/rodent TPPP is also present in mitochondrial membrane and sub-cellular mitochondrial fractions^11^. Interestingly, human TPPP show dual functions under physiological and pathological conditions^12^. Under physiological conditions, TPPP is expressed in differentiated oligodendrocytes and is critical for myelin sheath elongation^12–15^. However, under pathological conditions such as PD or Lewy body dementia, TPPP is present in neuronal inclusions in patient brains^3,5,6^.

Despite the initial report on the presence of TPPP in pathological aggregates in PD and related disorders more than a decade and half ago^3^, the role that TPPP specifically plays in the pathogenesis of these disorders remains to be elucidated. Recently, we identified the only *Drosophila* homolog of human TPPP named Ringmaker (Ringer) from a large-scale forward genetic screen^10^. *ringer* mutants display cytoskeletal and synaptic deficits as well as defects in axonal growth and regeneration^10,16,17^. While the role of Ringer during embryonic and larval stages are relatively characterized^10,16^, what role Ringer plays in adult *Drosophila* nervous system remains to be explored. Given the clinical relevance of human TPPP in PD and related disorders, we set out to investigate whether *Drosophila ringer* loss- and/or gain-of-functions might display behavioral and neuropathological characteristics similar to human PD.

Here, we report that adult *ringer* mutants show progressive locomotor decline, and reduced lifespan compared to their age- and gender-matched controls. We show that Ringer localizes to neuronal mitochondria and loss of Ringer display mitochondrial structural damage and dysfunctions. Our overall findings from *ringer* mutants resemble some of the salient features of human PD^2^. Interestingly, the locomotor deficits in *ringer* mutants were significantly improved upon treatment with L-DOPA, which is used in the clinical treatment of human PD. These findings allow us to utilize *ringer* mutants as an experimental fly model of PD and address the in vivo functions of TPPP. Together, our studies may provide key insights into the role of human TPPP in PD-related pathologies and lay the groundwork for a more comprehensive genetic and molecular analyses of TPPP.

## Results

### Adult *ringer* mutants show progressive locomotor deficits and reduced life span

**Figure 1:**
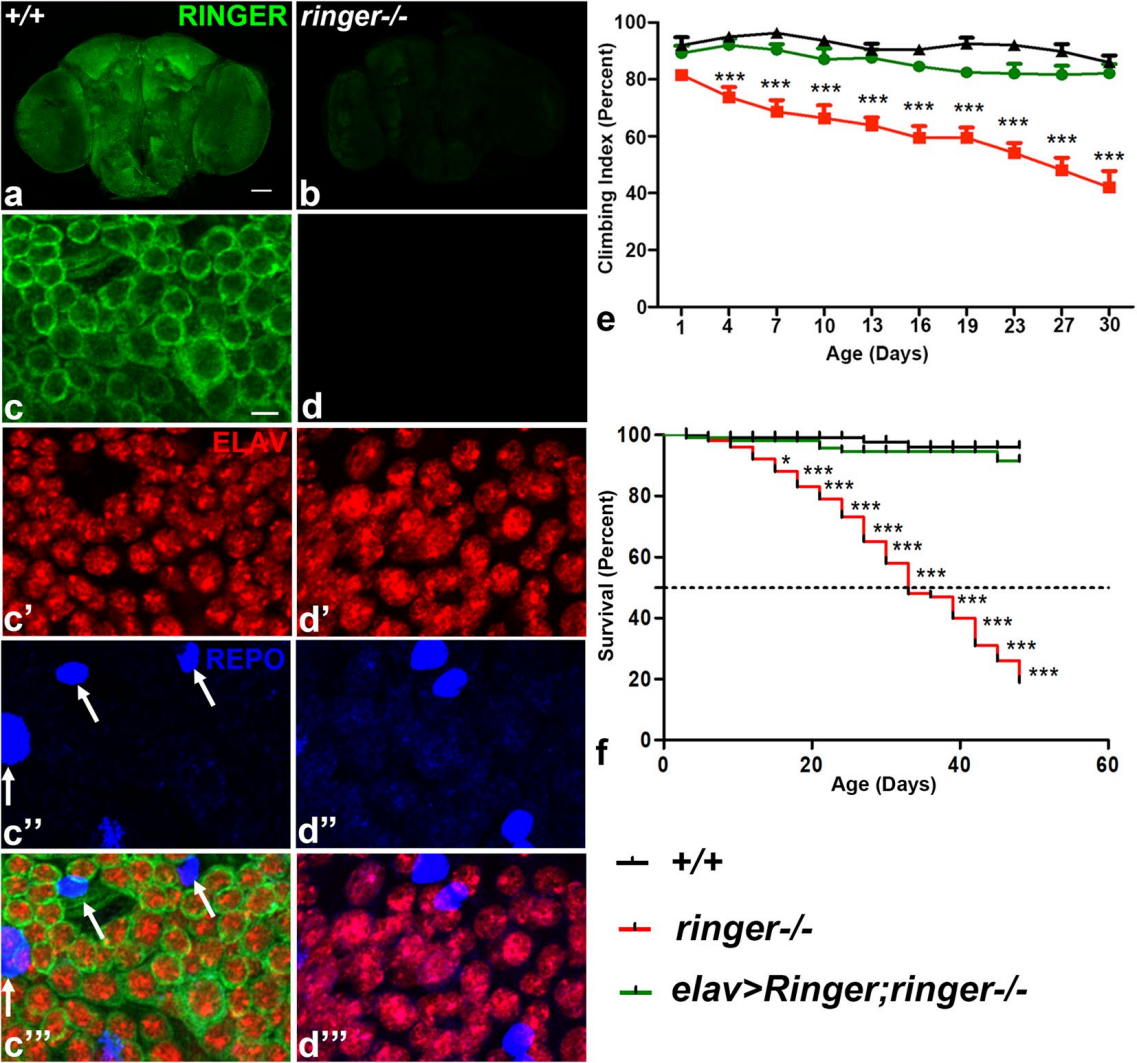
Ringer is expressed in neurons of adult *Drosophila* brain and loss of Ringer leads to locomotor disability and reduced lifespan. (**a**, **b**) Lower magnification confocal images of day 3 whole-mount adult brain of wild type (**a**) and *ringer*−/− (**b**) stained with anti-Ringer (green). (**c**-**d’’’**) Higher magnification confocal images of adult brains of wild type **c**–**c’’’** and *ringer*−/− (**d**-**d’’’**) stained with anti-Ringer (green), (**c**, **c’”** and **d**, **d’”**), anti-ELAV (red), (**c’**, **c’”** and **d’**, **d’”**) and anti-REPO (blue), (**c”**, **c’”** and **d”**, **d’”**). (**e**) Climbing ability of wild type (black), *ringer*−/− (red) and *elav* > *Ringer;ringer*−/− (green) rescue flies. n = 50 flies per genotype. Statistics was done using two-way ANOVA, *ringer*−/− ***p = 0.0001, *elav* > *Ringer;ringer*−/− *p* = 0.6883. (**f**) Lifespan analysis of wild type (black), *ringer*−/− (red) and *elav* > *Ringer;ringer*−/− (green) rescue flies. n = 200 flies per genotype. Kaplan-Meyer curve, log rank test, *ringer*−/− *** *p* < 0.0001, *elav* > *Ringer;ringer*−/− *p* = 0.317. Scale bars: (**a,b**) = 20 µm, (**c**-**d’”**) = 5 µm.

To investigate Ringer function in the adult nervous system, we first characterized the localization of Ringer in the adult brain. We immunostained wild type (+/+, Fig. 1a) and *ringer* mutant (*ringer*−/− , Fig. 1b) brains with anti-Ringer (green, Fig. 1a,b). Confocal image of a whole-mount adult brain at low magnification showed a widespread Ringer localization in wild type neurons (Fig. 1a), which was absent in the *ringer* mutants (Fig. 1b). Higher magnification confocal images from adult wild type (Fig. 1c-c’”) and *ringer* mutant (Fig. 1d-d’”) brains stained with anti-Ringer (green, Fig. 1c, c”’ and d, d’”) together with antibodies against a pan-neuronal nuclear protein, Elav (red, Fig. 1c’, c’” and d’, d’”) and a glial nuclear protein, Repo (blue, Fig. 1c”, c’” and d”, d’”) showed Ringer expression in neurons surrounding the Elav-positive cells in the wild type (Fig. 1c’, c”’) which was absent in *ringer* mutants (Fig. 1d’, d”’). Ringer expression could not be detected in the glial cells in wild type (arrows, Fig. 1c”, c’”). We next determined the functional consequences of loss- and gain-of Ringer in adult locomotor behavior and lifespan. *ringer* mutants (red, Fig. 1e) exhibited a progressive loss of locomotor abilities as seen from their climbing behavior (see Supplementary Movies) analyzed by rapid iterative negative geotaxis (RING) assay^18^ compared to age-matched controls (black, Fig. 1e). In addition, we also found that *ringer* mutants (red, Fig. 1f; Supplementary Fig. 1a) displayed reduced lifespan compared to the wild type age- and gender-matched controls (black, Fig. 1f and Supplementary Fig. 1a, respectively). However, there were no changes in the locomotor ability and lifespan in flies over-expressing Ringer in neurons as seen in *elav-Gal4; UAS-ringer* compared to age-matched wild type controls (Supplementary Fig. 1b-d). Interestingly, both the progressive locomotor deficits and reduced lifespan seen in *ringer* mutants could be fully rescued by expressing Ringer in all neurons, as seen in *elav-Gal4;UAS-ringer;ringer*−/− (green, Fig. 1e,f, respectively) rescue flies. These data indicate that Ringer function is required in neurons.

### *ringer* mutants show phenotypes associated with neurodegeneration

**Figure 2:**
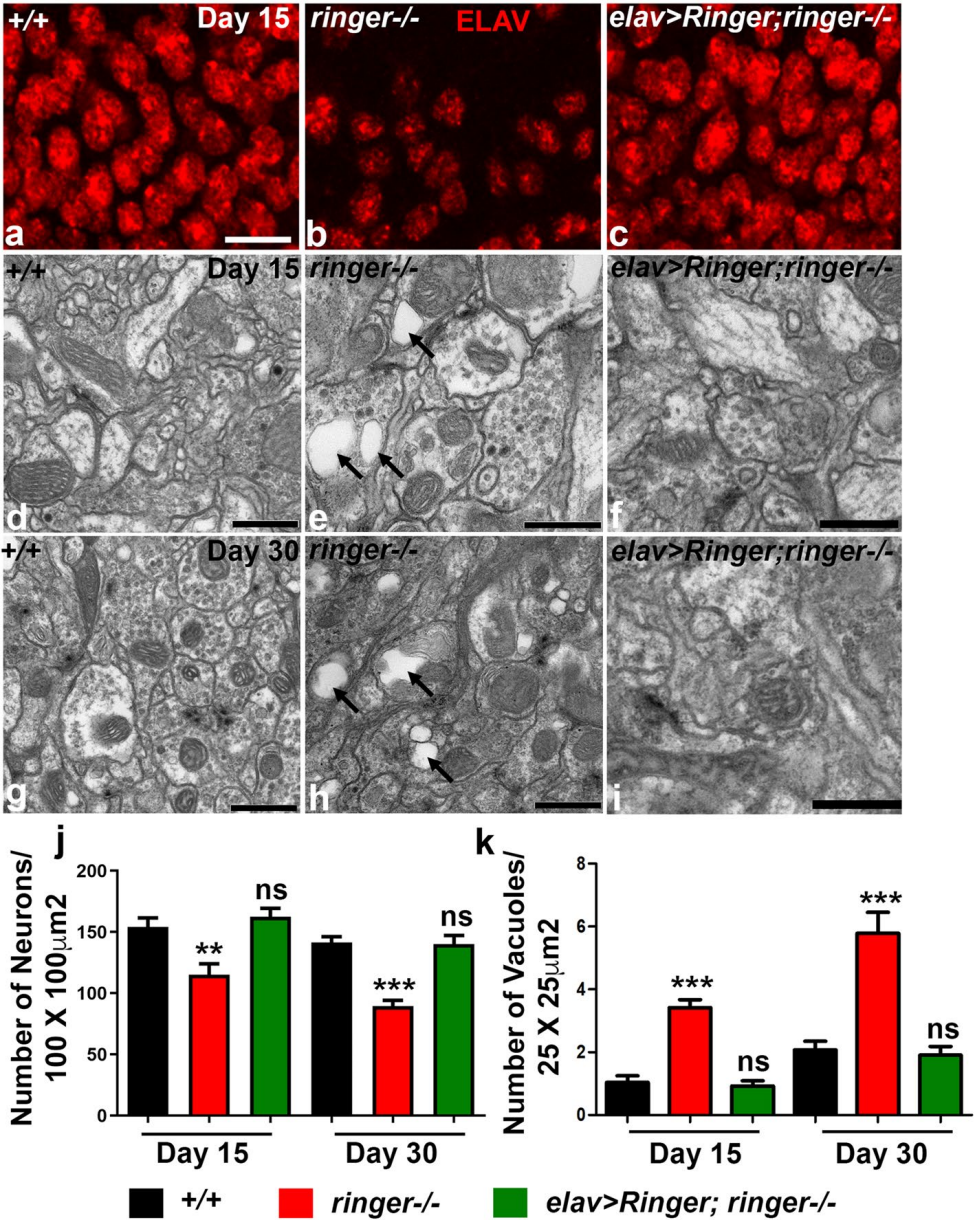
*Ringer* mutants show phenotypes consistent with neurodegeneration. (**a**-**c**) Confocal images of day 15 adult *Drosophila* of wild type (**a**), *ringer* mutant (**b**) and *elav* > *Ringer;ringer*−/− rescue (**c**) brains stained with anti-ELAV (red). (**d**-**i**) TEM images of day 15 (**d**-**f**) and day 30 flies (**g**-**i**) of wild type (**d**, **g**), *ringer*−/− (**e**, **h**) and *ringer* rescue (**f**, **i**)**.** (**j**) Quantification reveals a progressive decrease in number of neurons per 100 μm^2^ area from mushroom body regions of adult brains of day 15 and day 30 *ringer*−/− (red) compared to wild type (black) and *ringer* rescue flies (green). n = 7 brains per genotype. Statistics was done using one-way Anova with Tukey’s multiple comparison test, day 15 *ringer*−/− ** *p* = 0.0015, *ringer* rescue ns *p* = 0.7018, day 30 *ringer*−/− ***p = 0.0008, *ringer* rescue ns *p* = 0.3964. (**k**) Increased number of vacuoles in neurons of day 15 and day 30 *ringer*−/− (red) compared to wild type (black) and *ringer* rescue flies (green). n = 6 brains per genotype. Statistics was done using one-way Anova with Tukey’s multiple comparison test, day 15 *ringer*−/− ***p < 0.0001, *ringer rescue* ns *p* = 0.6763, day 30 *ringer*−/− ***p < 0.0001, *ringer rescue* ns *p* = 0.6825. Scale bars: (**a**-**c**) = 5 µm, (**d**-**f**, **i**) = 600 nm and (**g**, **h**) = 800 nm.

We next wanted to investigate the phenotypic consequences of loss of Ringer in adult *Drosophila* brain. Control and *ringer* mutant flies were analyzed at day 1, 15 and 30 to examine any given phenotype and assess if there is a progressive decline with age (Fig. 2 and Supplementary Fig. 2).We first examined the number of neurons as a measure of neuronal density in *ringer* mutants (Fig. 2b) quantified by counting of Elav-positive nuclei from an area of 100 × 100 µm^2^ imaged at 40× magnification from different brain regions such as mushroom bodies and subesophageal ganglia and compared them to comparable brain regions of age-matched wild type controls (Fig. 2a). While day 1 flies of *ringer* mutants and wild type did not show any significant difference in the number of neurons quantified (Supplementary Fig. 2a), day 15 and day 30 *ringer* mutants showed a significant and progressive decrease in the number of neurons (Fig. 2b,j) compared to age-matched wild type (Fig. 2a,j). Decreased neuronal density in *ringer* mutants were fully rescued to wild type levels when Ringer was expressed in neurons as seen in *elav-Gal4; UAS-Ringer; ringer*−/− (referred as *ringer* rescue) (Fig. 2c,j). Representative images from the mushroom body region of the adult day 15 brains from wild type (Fig. 2a), *ringer* mutants (Fig. 2b) and *ringer* rescue (Fig. 2c) are shown.

We next wanted to test *ringer* mutant brains for the presence of vacuoles using transmission electron microscopy (TEM). Presence of vacuoles is commonly associated with neurodegeneration in *Drosophila* as well as vertebrates^19,20^. Higher resolution TEM from *ringer* mutants of day 15 (Fig. 2e, quantified in k) and day 30 (Fig. 2h, quantified in k) showed presence of increased number of vacuoles (arrows, Fig. 2e,h) in brains analyzed from the mushroom body region when compared to wild type day 15 (Fig. 2d,k) and day 30 (Fig. 2g,k). There was no significant difference in the presence of vacuoles in young day 1 flies of wild type and *ringer* mutants (Supplementary Fig. 2b). Increased vacuolation in *ringer* mutants were fully rescued to wild type levels when Ringer was expressed in neurons as seen in *elav-Gal4; UAS-Ringer; ringer*−/− (Fig. 2f,i,k)*.* Together theses findings reveal that *ringer* mutants display decrease in neuronal density and increased vacuolation, phenotypes that are associated with neurodegeneration.

### Ringer loss leads to mitochondrial ultrastructural defects and Ringer is present in subcellular mitochondrial fractions

**Figure 3:**
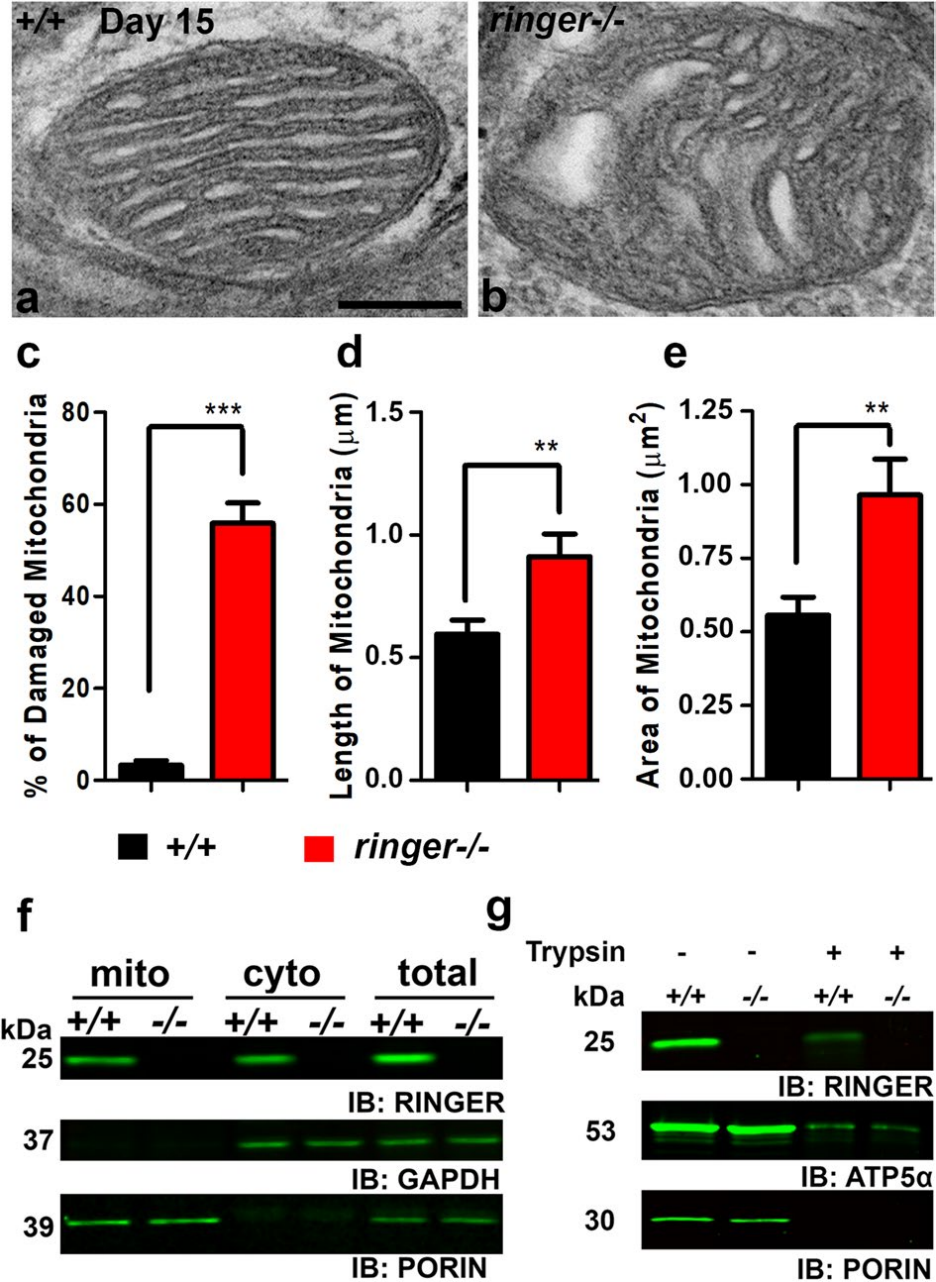
*Ringer* mutants show mitochondrial ultrastructural deficits and Ringer is detected in subcellular mitochondrial fractions. (**a**, **b**) Electron micrograph of mitochondria from day 15 flies shows enlarged mitochondria with disrupted cristae morphology in *ringer*−/− (**b**) compared to wild type (**a**). (**c**-**e**) Quantification of percentage of damaged mitochondria (**c**), mitochondrial length (**d**) and mitochondrial area (**e**) from ultrastructural analyses of wild type (black) and *ringer*−/− (red) mitochondria from mushroom body region of the adult brains. n = 6 brains per genotype. Statistics was done using unpaired student’s *t* test. (**c**) ***p < 0.0001, (**d**) **p = 0.0079, (**e**) **p = 0.0063. (**f**) Immunoblots of subcellular fractionation from wild type and *ringer*−/− fly heads probed for anti-Ringer, anti-Porin and anti-GAPDH in the mitochondrial (mito) and cytoplasmic (cyto) fractions and total lysates. (**g**) Immunoblots of anti-Ringer, anti-Porin and anti-ATP5α from trypsin-treated mitochondrial fractions of wild type and *ringer*−/− fly heads.

Apart from increased vacuolation, our TEM analysis in the preceeding section also indicated frequent anomalies in mitochondrial morphology in *ringer* mutants. Thus, we next wanted to analyze in more details the mitochondrial ultrastructure in *ringer* mutants compared to age-matched wild type controls from the mushroom bodies of the adult brains. TEM analysis of adult brains of wild type (Fig. 3a) and *ringer* mutants (Fig. 3b) of day 15 flies showed significantly disrupted mitochondrial cristae morphology in *ringer* mutants (Fig. 3b) compared to the wild type (Fig. 3a). In addition, *ringer* mutants also displayed significantly higher number of damaged mitochondria (Fig. 3c), increase in mitochondrial length (Fig. 3d) and area (Fig. 3e) compared to wild type (Fig. 3c-e, respectively). These findings reveal that loss of Ringer affects the stereotypic cristae morphology and the mitochondrial ultrastructure.

Presence of mitochondrial ultrastructural abnormalities in *ringer* mutants raised the possibility that Ringer might be associated with mitochondria. We next tested the presence of Ringer in mitochondrial fractions prepared from the adult *Drosophila* brain of wild type and *ringer* mutants (Fig. 3f, Supplementary Fig. 3a). We found that Ringer is present in both the mitochondrial and cytosolic fractions of wild type, and as expected, was absent in *ringer* mutants (Fig. 3f, Supplementary Fig. 3a). Anti-GAPDH, used as loading control (Fig. 3f, Supplementary Fig. 3a), was absent in the mitochondrial fractions, while Porin, a mitochondrial protein homologous to vertebrate VDAC^21,22^ (Fig. 3f, Supplementary Fig. 3a) was present in the mitochondrial fractions but excluded from the cytosolic fractions of wild type and *ringer* mutants. We next wanted to investigate if Ringer was associated with the outer or inner mitochondrial membrane. Wild type and *ringer* mutant mitochondrial fractions were treated with Trypsin (Fig. 3g) to eliminate any peripheral outer mitochondrial membrane proteins and mitochondrial protein precursors^23^. Western blot analysis of trypsin untreated (−) and treated (+) mitochondrial fractions detected the presence of Ringer, albeit at lower levels in trypsin-treated fractions of wild type (Fig. 3g, Supplementary Fig. 3b) suggesting that Ringer likely associates with the inner mitochondrial membrane. We used antibodies against ATP5α, a known inner mitochondrial membrane protein and Porin, which associates with the outer mitochondrial membrane as our controls (Fig. 3g, Supplementary Fig. 3b). ATP5α levels also showed a significant decrease upon trypsin treatment similar to what was observed with Ringer (Fig. 3g, Supplementary Fig. 3b). These data indicate that Ringer is present in mitochondrial subcellular fractions and might be associated with inner mitochondrial membrane.

### Ringer localizes to neuronal mitochondria and* ringer* mutants show abnormal mitochondrial morphology

**Figure 4:**
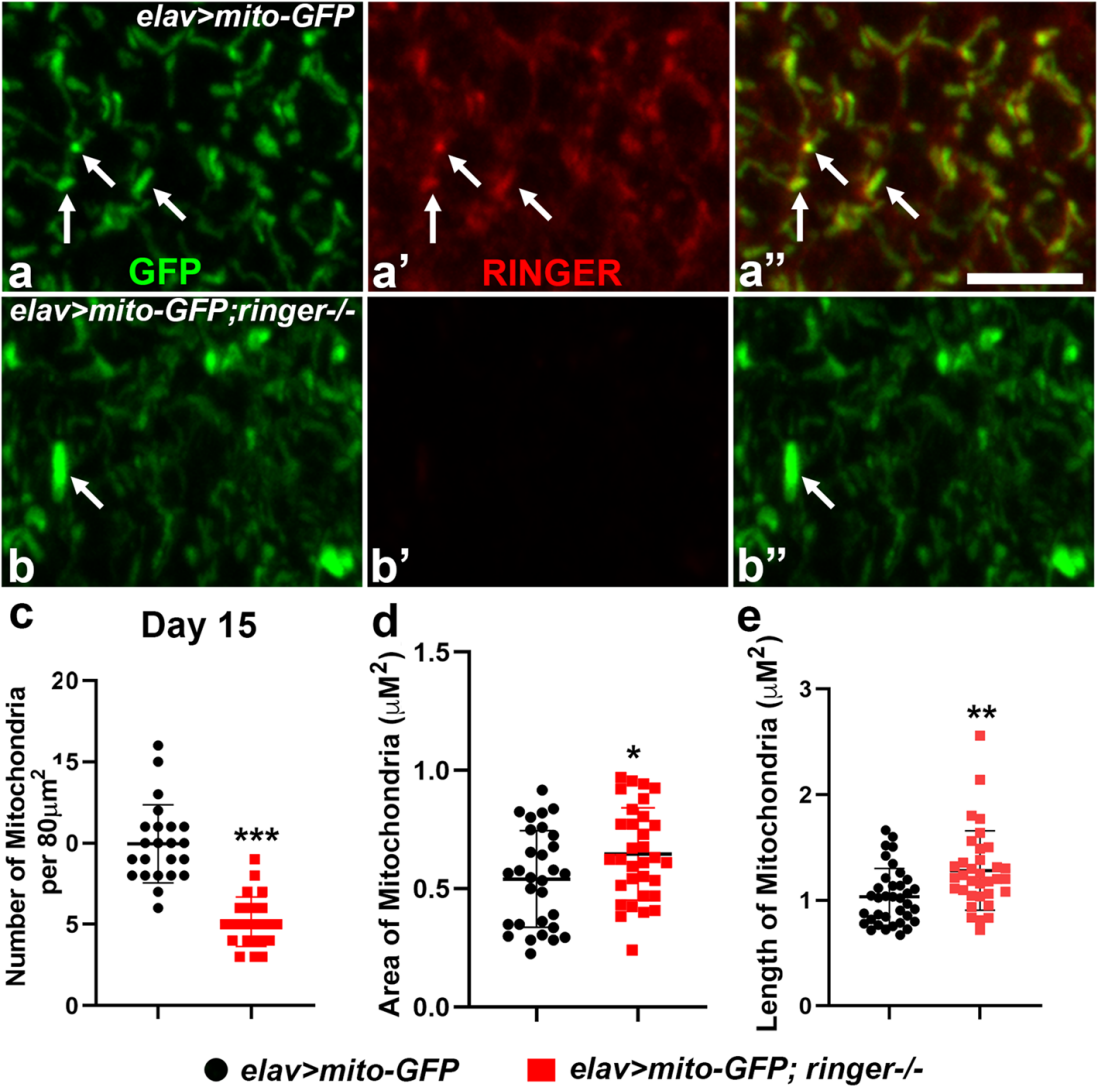
Ringer localizes to mitochondria and loss of Ringer leads to mitochondrial enlargement. (**a**-**b”**) Confocal images of day 15 adult brains stained with with anti-GFP (green), (**a**, **a”** and **b**, **b”**) and anti-Ringer (red), (**a’**, **a”** and **b’**, **b”**) in *elav* > *mito-GFP* (**a**-**a”**) and *elav* > *mito-GFP,ringer*−/− (**b**-**b”**). (**c**-**e**) Quantification of the number of mitochondria (**c)**, area of mitochondria (**d**) and length of mitochondria (**e**) in day 15 flies of *elav* > *mito-GFP* (black) and *elav* > *mito-GFP,ringer*−/− (red). Scale bar in (**a**-**b”**) = 5 µm. n ≥ 7 brains for each genotype. Statistics was done using unpaired student’s *t* test. (**c**) ***p < 0.0001, (**d**) * *p* = 0.038, (**e**) ** *p* = 0.0051.

Since Ringer was detected in subcellular mitochondrial fractions, we next wanted to examine if Ringer localized to mitochondria. We utilized a reporter line *UAS-mito-GFP* that targets GFP to mitochondria and first stained adult brains of *elav-Gal4;UAS-mito-GFP* to study Ringer localization with respect to GFP-labeled neuronal mitochondria (Fig. 4a-a”). A confocal section of the adult brain with punctate distribution of neuronal mitochondria (arrows, green, Fig. 4a,a”) showed significant overlap with Ringer (arrows, red, Fig. 4a’, a”) suggesting that Ringer localizes to neuronal mitochondria. As expected, Ringer was absent in *elav-Gal4; UAS-mito-GFP; ringer*−/− brain (Fig. 4b’, b”) while the GFP labeled mitochondria (arrow, Fig. 4b,b”) in *ringer* mutants showed multiple deficits compared to their respective controls (Fig. 4a-a”, c-e) resembling the ultrastructural deficits seen in the preceding section (Fig. 3). Among the various mitochondrial parameters analyzed, *ringer* mutant mitochondria when compared to corresponding age-matched controls at day 15 (Fig. 4c-e) and day 30 (Supplementary Fig. 4a-c) showed significant decrease in the total number of mitochondria (Fig. 4c, Supplementary Fig. 4a), and a significant increase in the mitochondrial area (Fig. 4d; Supplementary Fig. 4b) as well as mitochondrial length (Fig. 4e, Supplementary Fig. 4c). These data show that Ringer localizes to neuronal mitochondria in the adult brain and loss of Ringer leads to defects in mitochondrial morphology.

### Loss of Ringer leads to mitochondrial dysfunctions

**Figure 5:**
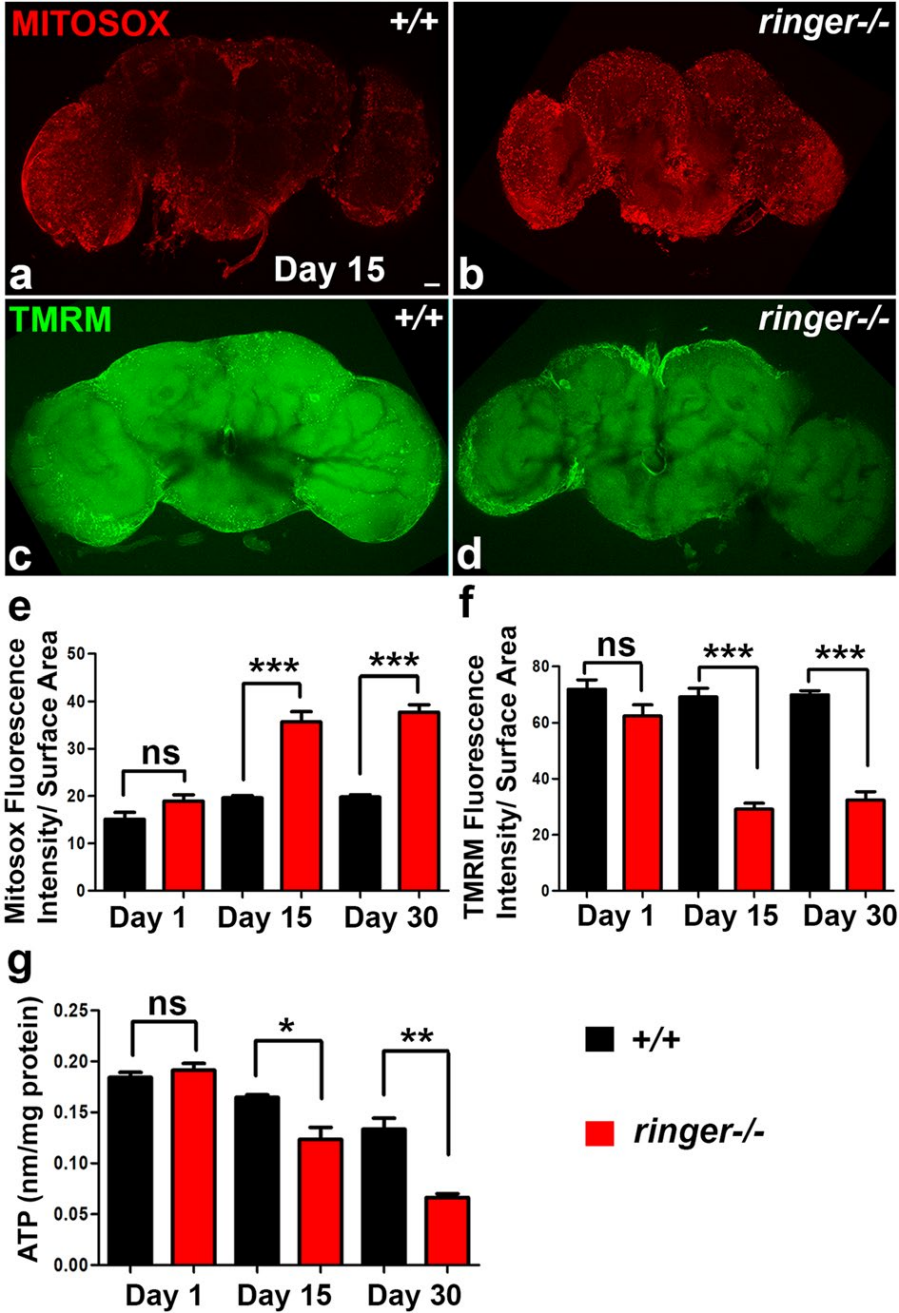
*Ringer* mutants show mitochondrial dysfunctions. (**a**, **b**) MitoSOX red fluorescence in day 15 whole-mount adult brain preparations from wild type (**a**) and *ringer*−/− (**b**) flies. (**c**, **d**) TMRM green fluorescence in day 15 whole-mount brain preparations from wild type (**c**) and *ringer*−/− (**d**) flies. (**e**, **f**) Quantification of fluorescence intensity of MitoSOX (**e**) and TMRM (**f**) normalized to the surface area of the brains from wild type (black) and *ringer* mutants (red) from day 1, 15 and 30 flies. Scale bar in (**a**-**d**) = 20 µm and n = 7 brains per genotype. (**g**) Analysis of ATP levels in day 1, day 15 and day 30 flies of wild type (black) and *ringer*−/− (red). n = 3 (each experiment was done independently three times). Statistics were done using unpaired student’s *t* test. (**e**) day1 *p* = 0.0896, day15 ***p < 0.0001, day30 ***p < 0.0001, (**f**) day1 ns *p* = 0.1086, day15 ***p < 0.0001, day30 ***p < 0.0001 and (**g**) day 1 ns *p* = 0.4445, day15 *p = 0.0283, day30 **p = 0.0044.

The mitochondrial structural abnormalities in *ringer* mutants raised the possibility of mitochondrial dysfunctions, we next investigated for any mitochondrial functional changes in *ringer* mutants. We analyzed the superoxide levels in the adult wild type and *ringer* mutant brains by using MitoSOX dye (Fig. 5a,b, respectively, quantified in e) and also performed tetramethylrhodamine methyl ester (TMRM) assays (Fig. 5c,d, respectively, quantified in f) to measure the mitochondrial membrane potential (MMP). We used freshly dissected whole mount wild type and *ringer* mutant brains of day 1, 15 and 30 flies for these assays. MitoSOX upon permeating live cells gets targeted to mitochondria and becomes oxidized by superoxide resulting in red fluorescence which can be measured by confocal microscopy^24^. The TMRM dye is a cell-permeant dye which can accumulate in healthy mitochondria with intact MMP, and the fluorescence intensity drops dramatically in dysfunctional mitochondria^25^. While day 1 *ringer* mutants showed no significant differences in MitoSOX (Fig. 5e) or TMRM (Fig. 5f) intensities compared to wild type, day 15 and 30 adult brains analyzed for MitoSOX fluorescence intensity revealed significantly elevated levels in *ringer* mutants (Fig. 5b,e) compared to wild type (Fig. 5a,e) suggesting elevated levels of mitochondrial superoxide production and oxidative stress. TMRM fluorescence intensity, on the other hand, displayed a significant reduction in day 15 and 30 *ringer* mutant flies (Fig. 5d,f) compared to wild type (Fig. 5c,f) suggesting a decrease in MMP and depolarization of *ringer* mutant mitochondria. We next measured the ATP levels^26^, which is a measure of mitochondrial function, in wild type and *ringer* mutant day 1, 15 and 30 flies and observed that day 15 and 30 *ringer* mutant flies showed significantly decreased amount of ATP compared to the age-matched wild type flies (Fig. 5g). Day 1 flies of *ringer* mutants did not show any significant difference in any of the mitochondrial functional parameters assessed compared to wild type day 1 flies (Fig. 5e-g). These data show that Ringer loss causes mitochondrial functional changes leading to increased mitochondrial superoxide levels as well as decreased MMP and ATP levels.

### *ringer* mutants are susceptible to mitochondrial toxin, Rotenone, and show improvements when treated with NAC

**Figure 6:**
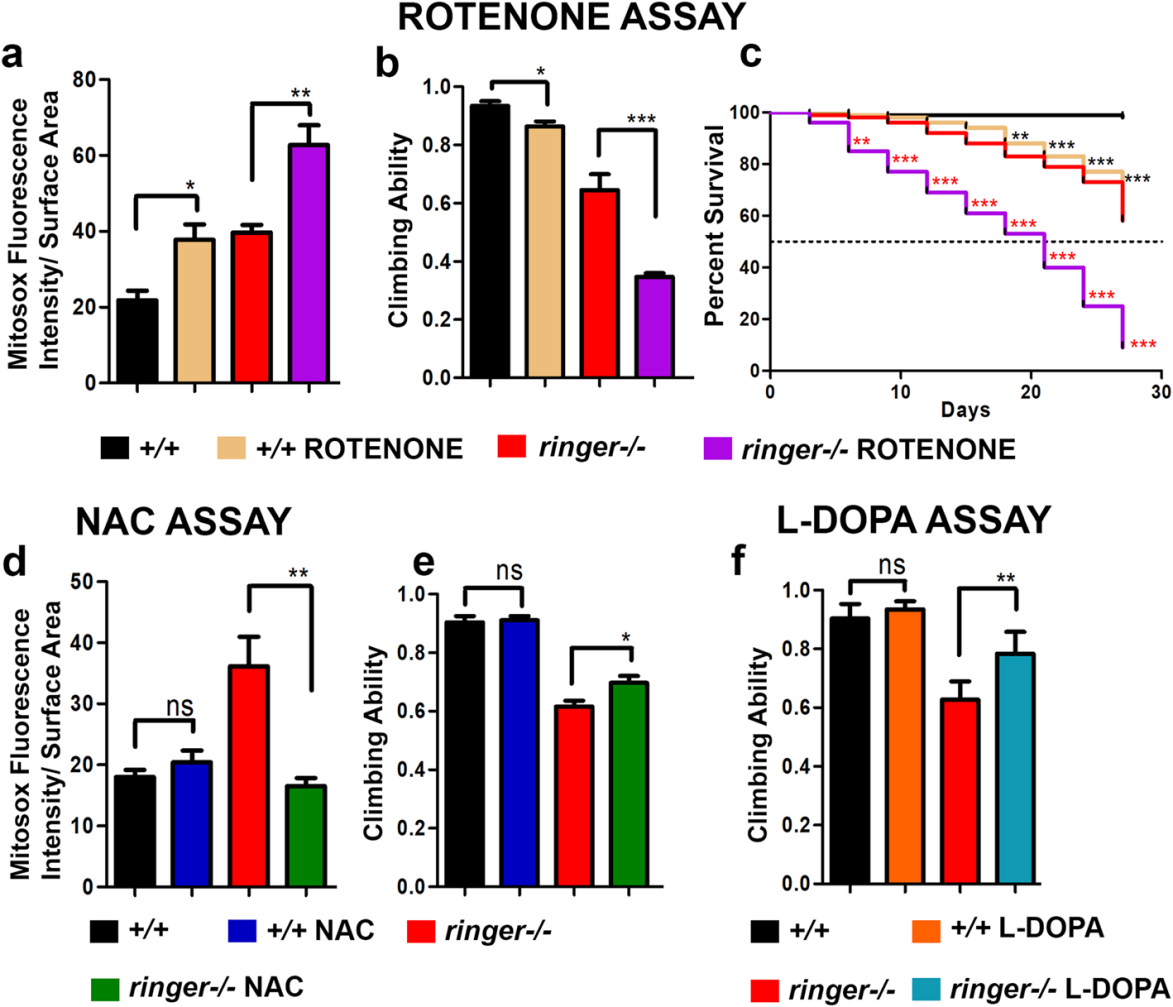
*Ringer* mutants are susceptible to mitochondrial toxin, rotenone, and show improvement in behavior when treated with L-DOPA. (**a**-**c**) Quantification of MitoSOX red fluorescence intensity (**a**), climbing ability (**b**) and life span analysis (**c**) of wild type untreated (black), wild type treated with rotenone (brown), *ringer*−/− untreated (red) and *ringer*−/− treated with rotenone (purple). (**d**, **e**) Quantification of MitoSOX red fluorescence intensity (**d**) and climbing ability (**e**) of wild type untreated (black), wild type treated with NAC (blue), *ringer*−/− untreated (red) and *ringer*−/− treated with NAC (green). (**f**) Quantification of climbing ability of wild type untreated (black), wild type treated with L-DOPA (orange), *ringer*−/− untreated (red) and *ringer*−/− treated with L-DOPA (teal). n = 7 brains per genotype for MitoSOX assay; n = 50 flies for climbing assay; and n = 200 flies for life span analysis. Statistics were done using unpaired student’s *t* test for quantification of MitoSOX fluorescence intensity and climbing ability. Log rank test was used for life span analysis. (**a**) *p = 0.0124, **p = 0.0063. (**b**) *p = 0.0368, ***p = 0.0001. (**c**) +*/*+ untreated with +/+ treated with rotenone, *** *p* < 0.0001, *ringer*−/− untreated with *ringer*−/− treated with rotenone, ***p < 0.0001. (**e**) +/+  with +/+  treated with NAC *p* = 0.2840, **p = 0.0010. (**d**) +/+  with +/+ treated with NAC *p* = 0.8201, *p = 0.0418. (**e**) +/+  with +/+ treated with L-DOPA *p* = 0.3671, **p = 0.0062.

Mitochondrial functions are often characterized by the various protein complexes that drive the electron transport chain and generation of ATP. We wanted to determine whether a well-characterized mitochondrial toxin such as rotenone affected *ringer* mutant mitochondria and to what extent compared to the wild type mitochondria? Rotenone is a known toxin and its exposure models PD by inhibiting mitochondrial Complex I^27^. After feeding rotenone at similar concentration and length of time, both wild type and *ringer* mutant flies showed significantly higher mitochondrial superoxide levels (Fig. 6a), impaired locomotor abilities (Fig. 6b) and reduced life span (Fig. 6c) compared to their respective untreated counterparts (Fig. 6a-c). However, compared to the rotenone-treated wild type flies, rotenone-treated *ringer* mutant flies were far more susceptible to rotenone as they showed significantly increased severity in all 3 phenotypic categories analyzed (Fig. 6a-c). Altogether, these data indicate that *ringer* mutant flies are more susceptible to the mitochondrial toxin and PD stressor, rotenone, implicating a compromised Complex I activity in *ringer* mutants.

We next wanted to investigate what response *ringer* mutant flies have upon treatment with the reactive oxygen species (ROS) scavenging antioxidant, N-Acetyl-L-cysteine (NAC). NAC can increase glutathione stores to enhance clearance of mitochondrial ROS and can also chemically reduce ROS^28,29^. Following a 2-weeks long treatment with NAC, the mitochondrial ROS levels in *ringer* mutant flies returned to normal wild type levels (Fig. 6d). We also observed a significant improvement of locomotor abilities in NAC-treated *ringer* mutant flies compared to untreated *ringer* mutants (Fig. 6e). However, this improvement in locomotor ability did not achieve wild type levels. Wild type NAC-treated flies did not show any difference in ROS levels (Fig. 6d) or locomotor behavior (Fig. 6e) when compared to NAC-untreated wild type counterparts. Feeding with NAC also did not result in increased longevity of the *ringer* mutant flies (Supplementary Fig. 5a). These findings highlight that NAC treatment effectively reduced ROS levels in *ringer* mutants.

### Treatment of* ringer* mutants with L-DOPA improves locomotor behavior

We next wanted to utilize a pharmacological agent that is unrelated to mitochondrial functions but has been used as a treatment option for human PD, which is the DA-precursor, 3,4-dihydroxyphenylalanine (L-DOPA)^30,31^, in order to examine any improvement of *ringer* mutant phenotypes. More specifically, we were interested in examining if the locomotor ability of the *ringer* mutants could be ameliorated by feeding L-DOPA. Following L-DOPA treatment for 2 weeks, the locomotor ability of *ringer* mutants was indeed significantly improved compared to untreated *ringer* mutants (Fig. 6f). However, the mobility of the L-DOPA treated *ringer* mutants could not be restored to control wild type flies of same age. As expected, the lifespan and the mitochondrial superoxide levels seen in *ringer* mutants treated with L-DOPA did not improve compared to untreated *ringer* mutants (Supplementary Fig. 5b, c, respectively). These data show that locomotor behavior of *ringer* mutants can be significantly improved by treatment with L-DOPA.

### Ringer mutant flies display progressive loss of dopaminergic neurons

**Figure 7:**
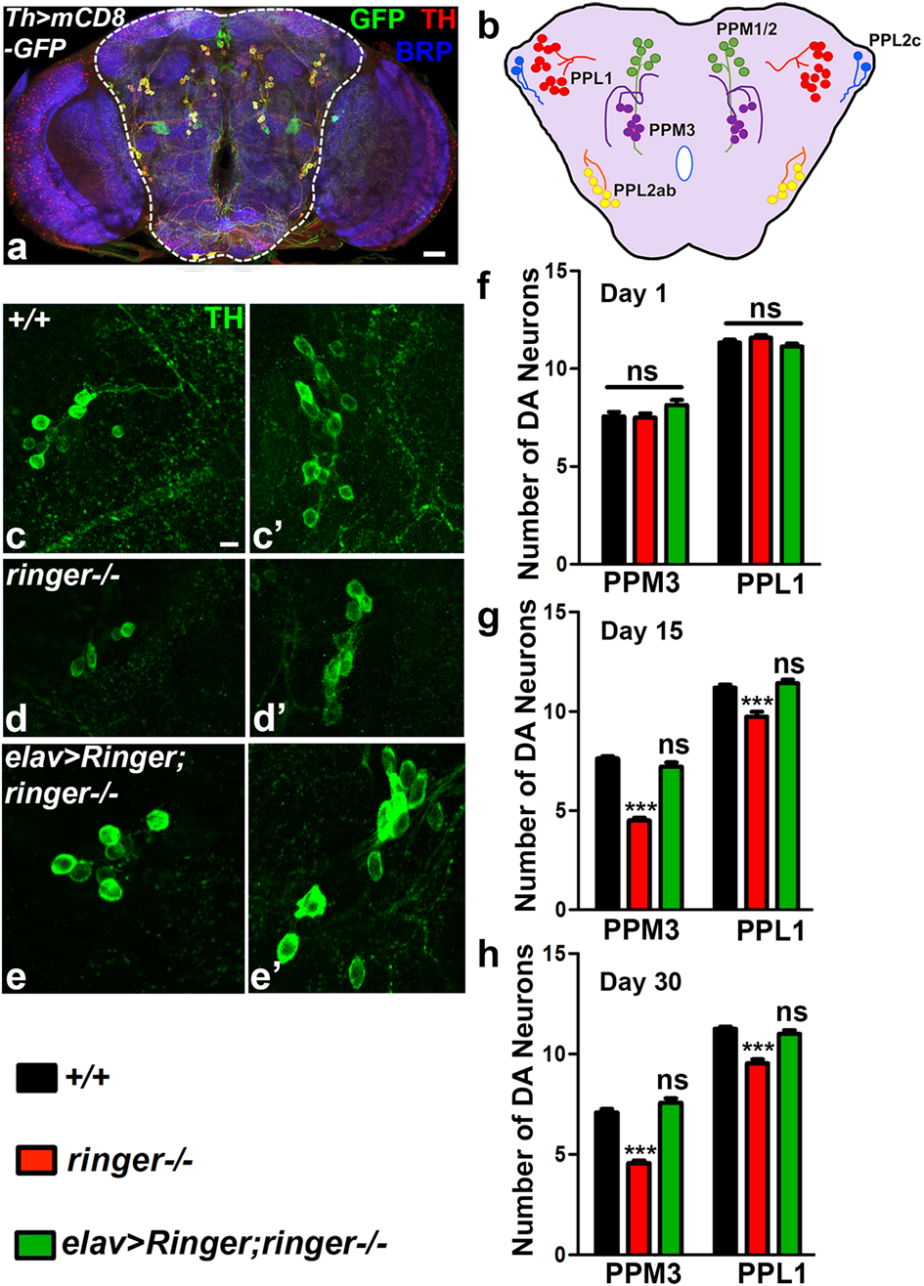
*Ringer* mutants show progressive loss of dopaminergic neurons. (**a**-**b**) Adult brain immunostaining (**a**) with anti-TH (red), anti-GFP (green) and anti-Brp (blue) of *Th* > *mCD8-GFP* and corresponding schematic (**b**) showing distinct DA neuron clusters on the posterior brain. (**c**-**e’**) Higher magnification confocal images of PPM3 (**c**-**e**) and PPL1 (**c’**-**e’**) clusters stained with anti-Th (green) of day 15 wild type (**c**, **c’**), *ringer*−/− (**d, d’**), and *ringer* rescue as seen in *elav* > *Ringer;ringer*−/− (**e**, **e’**). (**f**–**h**) Quantification of numbers of DA neurons in specified clusters of day 1 (**f**), day 15 (**g**) and day 30 (**h**) flies per brain hemisphere. n = 12 brains (~ 24 brain hemispheres) for each genotype. Statistics was done using two-way ANOVA. (**f**) day 1 PPM3 *ringer*−/− *p* = 0.9789, *elav* > *Ringer;ringer*−/− *p* = 0.7930, PPL1 *ringer*−/− *p* = 0.9509, *elav* > *Ringer;ringer*−/− *p* = 0.9958. (**g**) day 15 PPM3 *ringer*−/− ***p < 0.0001, *elav* > *Ringer;ringer*−/− *p* = 0.4653, PPL1 *ringer*−/− ***p < 0.0001, *elav* > *Ringer;ringer*−/− *p* = 0.9969. (**h**) day30 PPM3 *ringer*−/− ***p < 0.0001, *elav* > *Ringer;ringer*−/− *p* = 0.3161, PPL1 *ringer*−/− ***p < 0.0001, *elav* > *Ringer;ringer*−/− *p* = 0.7670. Scale bar: (**a**) = 20 µm, (**c**-**e’**) = 5 µm.

The improvement of locomotor deficits of *ringer* mutant flies by treatment with L-DOPA raised the possibility that *ringer* mutants may undergo loss of dopaminergic neurons (DA) resulting in reduced dopamine levels. We therefore analyzed *ringer* mutants for potential loss of DA neurons. The position and arrangement of DA neurons in the adult *Drosophila* brain has been well-documented^32,33^ and they appear as prominent clusters when labeled with antibodies against Tyrosine hydroxylase (anti-Th) (Fig. 7a). DA neuron clusters in the posterior brain are simplified in the schematic (Fig. 7b). We stained adult brains of wild type (Fig. 7c,c’), *ringer* mutants (Fig. 7d,d’) and *ringer* rescue as seen in *elav-Gal4; UAS-ringer; ringer*−/− (Fig. 7e, e’) with anti-Th (green, Fig. 7c-e’). We analyzed distinct DA neuron clusters in each brain hemispheres of the specified genotypes including the anterior PAL, T1 and Sb clusters, and the posterior PPM1, PPM2, PPM3, PPL1, PPL2ab and PPL2c clusters^33^. Young *ringer* mutants (day 1) did not show any significant loss of DA neuron clusters compared to wild type (Fig. 7f, Supplementary Fig. 6a). The onset of the DA neuronal loss in *ringer* mutants is seen around day 7 (Supplementary Fig. 6b) with PPM3 being the cluster getting affected first. *ringer* mutant day 15 flies (Fig. 7c, c’, g; Supplementary Fig. 6c) showed loss of PPM3 and PPL1 clusters of DA neurons compared to age-matched controls (Fig. 7c, c’, g; Supplementary Fig. 6c) indicating a progressive loss of DA neuron clusters in *ringer* mutants is progressive. It is also important to note that both PPM3 and PPL1 clusters in *Drosophila* are implicated in regulating locomotion^32^. Interestingly, the extent of the loss of DA neurons in day 30 *ringer* mutants increased within the same clusters (Fig. 7h) together with more clusters showing loss of DA neurons (such as PPM2, Supplementary Fig. 6d) compared to the corresponding wild type controls (Fig. 7h; Supplementary Fig. 6d). Loss of DA neurons in *ringer* mutants analyzed at different time points were fully rescued by expression of Ringer in neurons as seen in *elav-Gal4;UAS-ringer;ringer*−/− (Fig. 7e, e’, g, h and Supplementary Fig. 6b-d). These studies reveal that *ringer* mutants display loss of DA neurons.

## Discussion

In this study, we report the characterization of *Drosophila* TPPP, Ringer, in the adult nervous system. Our findings reveal that *ringer* mutants display progressive loss of locomotor abilities, mitochondrial structural damage and dysfunctions with increased ROS levels and altered MMP, as well as susceptibility to mitochondrial toxin, rotenone and loss of DA neurons. The phenotypes seen in *ringer* mutants resemble some of the characteristics of human PD. Thus, we propose that *ringer* mutants could serve as a fly model of human PD. Interestingly, development of simple genetically tractable models, such as *Drosophila,* has contributed enormously towards understanding human disease process and have emerged as a valuable model system for studying mechanisms of neurodegeneration underlying various neurodegenerative diseases^24,34^.

Although *ringer* mutants are not 100% viable since ~ 30% lethality is observed between embryonic to larval stages and another ~ 30% from larval to eclosion of adults (data not shown), our findings from the present study indicate that adult phenotypes resulting from loss of Ringer are mostly degenerative and not developmental. While young *ringer* mutant flies of day 1 do not typically show any of the phenotypic consequences that manifest after a week or more in adulthood, most phenotypes of adult *ringer* mutants become progressive with age. Moreover, we also observed that loss-of Ringer seemed more detrimental to the flies than gain-of Ringer functions (Fig. 1, Supplementary Fig. 1). One of the possibilities could be that wild type endogenous Ringer expresses abundantly in the neurons, thus, any further elevation of Ringer levels in neurons may not lead to any detrimental effects. Not surprisingly, loss of Ringer from neurons displayed phenotypes consistent with neurodegeneration (Fig. 2). General neurodegeneration as well as selective vulnerability of neurons is characteristic of human PD^35,36^ as well as other disorders including Alzheimer’s disease^37,38^.

Our findings on mitochondrial ultrastructural abnormalities (Fig. 3), Ringer localization in mitochondria (Fig. 4) and mitochondrial dysfunctions (Fig. 5) resulting from loss of Ringer all point towards an important role of Ringer in mitochondria. Detection of Ringer in Trypsin-treated mitochondrial fractions (Fig. 3) imply that Ringer most likely associates with the inner mitochondrial membrane. However, Ringer association with outer mitochondrial membrane cannot be ruled out. Since levels of Ringer following trypsinization was markedly decreased, it raises a possibility that Ringer might also associate with the outer mitochondrial membrane. The disrupted cristae morphology in *ringer* mutants also support the association of Ringer with inner mitochondrial membrane as it indicates that mitochondrial cristae might be a site of Ringer action, either directly or indirectly. The elaborate membrane architecture of cristae in an otherwise normal mitochondria is a prerequisite for efficient respiration and ATP generation as it harbors complexes of the electron transport/respiratory chain. Dysfunctional mitochondrial respiratory chain, particularly a deficit in Complex I activity is implicated in PD^1^. Complex I is a major source of superoxide production in the electron transport chain^39^. Mitochondrial dysfunctions can lead to increased levels of ROS, an important factor linked to PD pathogenesis and neuronal death^29,40^. Apart from Complex I, ROS production is also linked to Complex III^41^. Loss of Ringer displayed increased ROS levels (Fig. 5) indicative of mitochondrial dysfunction and a possible deficit in Complex I and/or Complex III activities. Similar sensitivity to ROS has also been reported in flies lacking Pink1^26^, Parkin^42^, and in fly models of a-Syn^24^ among other genes. The formation of pathological brain inclusions in PD and related disorders has been linked with energy deficiency, and the extent of the polarization of the mitochondrial membrane is an indication of the energy state of the cells, as determined by the function of the mitochondrial electron transport system^43^. *ringer* mutants seem to display deficits on both counts: (1) lower ATP levels reflective of energy deficiency, and (2) less TMRM fluorescence suggestive of defects in MMP. These mitochondrial anomalies in *ringer* mutants might be a causative factor for neurodegeneration.

Pharmacological assays (Fig. 6) performed on *ringer* mutants highlighted interesting features of Ringer function. Chronic administration of the environmental mitochondrial toxin, rotenone, is reported to cause selective degeneration of nigral dopaminergic neurons with pathological signature of PD and PD-like locomotor symptoms in animal models^44^. Consistent with these findings, *ringer* mutants also displayed increased susceptibility to rotenone and enhanced PD-like behavioral and pathological hallmarks, including increased locomotor deficits and elevated ROS levels, respectively. Exposure to rotenone is implicated in impaired Complex I activity in the mitochondrial respiratory chain leading to reduced ATP levels and elevated ROS^28,44^. These findings further provide credence to the possibility of a compromised Complex I activity in *ringer* mutant mitochondria. While rotenone treatment increased mitochondrial ROS and compromised locomotor behavior in *ringer* mutants, an opposite effect was observed upon treatment with the antioxidant, NAC. A clearance of mitochondrial ROS was observed upon NAC treatment of *ringer* mutants which may improve mitochondrial functions and a partial restoration of locomotor behavior indicating that ROS modulation might influence locomotor behavioral performance. Similar influence on cognitive behavioral performance by ROS modulation has been recently reported^29^.

The pharmacological intervention by feeding L-DOPA to *ringer* mutant flies (Fig. 6) resembled the treatment paradigm and outcome seen in human PD patients^30,31,45^. In the human PD patient brain, L-DOPA gets decarboxylated to dopamine and stimulates the dopaminergic receptors, thereby compensating for the depleted supply of endogenous dopamine. While motor symptoms of PD patients are improved upon treatment with L-DOPA, loss of neurons is not prevented^46,47^. The improvement of locomotor deficits of *ringer* mutant flies by L-DOPA treatment together with the progressive loss of DA neurons seen in *ringer* mutants are some of the striking features characteristic of human PD. In PD, selective vulnerability in DA neurons is also observed^36,48^ in addition to degeneration of many non-DA neurons as well as synapses^35,49^. Although the exact mechanism(s) of this selective neuronal vulnerability in some of the neurodegenerative disorders remains to be elucidated, there are many factors implicated in this pathology. These include specific combinations of genetic predispositions and environmental stressors, increased mitochondrial oxidative stress, a lack of calcium buffering proteins or disturbed cellular calcium regulation, all of which could trigger age-related stress and proteostasis dysfunctions in vulnerable neurons^36,38^. The ringer mutants could serve as an experimental system to further address the DA neuron loss as *ringer* mutants age and will provide insights as to which cellular processes lead to DA neuron loss in *ringer* mutants.

Taken together, the present study on the *Drosophila* TPPP/Ringer provides an entry point for further elucidating the role of TPPP in neurodegeneration. Our studies have laid the groundwork to further dissect the molecular processes that underlie the mitochondrial abnormalities seen in *ringer* mutants. As Ringer is a microtubule associated protein, its involvement in cytoskeletal functions coupled with its mitochondrial functions, it may play a much broader role in neuronal function and survival. What ultimately leads to neuronal degeneration and motor deficits in *ringer* mutants may provide windows of opportunity to gain insights into the role of human TPPP in neurodegeneration which is highly relevant to human PD and associated disorders.

## Methods

### Drosophila lines

The *Drosophila* lines used in this study include *w*^*1118*^ (wild type control), *elav-Gal4* (BDSC# 458), *Th-Gal4* (BDSC# 8448) and *UAS-mito-GFP* (BDSC# 8442) obtained from the Bloomington Drosophila Stock Center, Indiana. *ringer*^*915*^ and *UAS-ringer* were described previously^10,16^. All fly stocks were maintained at 25 °C, 50% humidity and with a 12-h light/dark cycle.

### Lifespan and locomotor assays

For lifespan assay, 20 flies were aged per vial and a total of 200 flies per genotype were analyzed for both males and females separately. Flies were transferred to fresh food every third day and maintained at 25 °C. Mortality was plotted using Kaplan-Meyer analysis in GraphPad Prism software.

To determine the climbing ability of the flies, RING assay^18^ was performed with slight modifications. Briefly, 10 freshly eclosed male flies were collected in individual vials and a total of 50 flies for each genotype were analyzed. The assay was started 24 h after CO_2_ anesthesia. Flies were gently tapped down to the bottom of an empty clear vial and the number of flies crossing a 10 cm mark drawn from the base of the vial within 10 s were recorded. Each assay was repeated 6 times with a recovery time of 1 min in between, and the mean and standard error of the mean was calculated. Flies were maintained at 25 °C for the entire duration of the assay.

### Immunohistochemistry and confocal microscopy.

Immunostaining of whole-mount adult fly brains was performed as previously described^50^. Dissection of adult brains was done in ice-cold PBS and fixed in 4% paraformaldehyde (PFA) prepared in PBS for 20 min. Brains were mounted in Fluoromount-G mounting medium (Southern Biotechnology, 0100–01). Primary antibodies used were anti-Ringer (1:750)^10^, anti-Elav (1:500, DSHB, 9F8A9), anti-Brp (1:250, DSHB, NC82), anti-Th (1:300, Novus Biologicals, NB300-109), anti-GFP (1:500, Invitrogen, A10262), and anti-Repo^51^. Secondary antibodies conjugated to Alexa 488, 568 and 647 (Invitrogen-Molecular Probes) were used at 1:400 dilution. Confocal images of all genotypes of the brains belonging to the same experimental group were acquired using the same settings with a Zeiss LSM710 confocal microscope, and image editing was done using Adobe Photoshop software.

### Electron microscopy

Ultrastructural analyses of age-matched adult fly heads of various genotypes were processed for TEM as previously described^52^ with minor modifications. Briefly, adult fly heads were fixed in 4% PFA/1% glutaraldehyde in 0.1 M cacodylic acid, pH 7.2 for 30 min at room temperature followed by overnight fixation at 4 °C. The fixed fly heads were rinsed in 0.1 M cacodylic acid, pH 7.2 and postfixed in 2% aqueous osmium tetroxide for 1 h, followed by rinsing and dehydration in increasing ethanol concentration. Samples were incubated for an hour in propylene oxide and gradually infiltrated in increasing resin to propylene oxide ratio (1:2, overnight; 2:1, 6 h; and full resin for 36 h with constant agitation). Samples were embedded in flat silicone molds with Polybed resin and cured in oven at 55 °C for 36 h. 7 male and female flies/genotype were processed for TEM analysis. The number of mitochondria (n) analyzed for each genotype was at least 50. Image J was used for quantification of various mitochondrial parameters described.

### Immunoblotting

Adult fly heads were used for immunoblotting according to previously published protocols^16^. The supernatants with equal amounts of proteins from each genotype were separated on SDS-PAGE for immunoblotting with respective antibodies. Each experiment was done independently three times. Primary antibodies used for immunoblotting were guinea pig anti-Ringer (1:10,000)^10,16^, anti-Porin (1:1000, Abcam, ab14734), anti-GAPDH (1:5000, Invitrogen, MA5-15,738-BTIN), anti-ATP5α (1:5000, Abcam, ab14748). Original unprocessed blots probed for all antibodies in Fig. 3 are provided in the Supplemental Information file (Supplementary Fig. 3), including full length blots for anti-Ringer, anti-GAPDH and anti-Porin shown in Supplementary Fig. 3a and anti-ATP5α in Supplementary Fig. 3b. Anti-Ringer and anti-Porin blots from Fig. 3b are shown as partial blots in Supplementary Fig. 3b as they were cut prior to hybridization to probe for other antibodies.

### Mitochondrial fractionation

Mitochondrial isolation from adult *Drosophila* heads was performed as previously described^53^ with few modifications. Briefly, adult fly heads were homogenized in mitochondrial fraction buffer (250 mM Sucrose, 10 mM Tris–HCl, 1 mM EGTA, pH = 7.5) with protease inhibitor cocktail (Sigma, 11,836,170,001). The homogenate was centrifuged at 800xg to pellet debris, and the supernatant collected and centrifuged at 10,000 g to yield a pellet containing mitochondria and supernatant containing cytoplasmic proteins. Three independent fractionation procedures were carried out and subcellular fractions were subjected to immunoblotting with respective antibodies.

### Trypsin treatment

Trypsin treatment was adapted from previously published method^23^. Isolated mitochondrial fractions were digested with 1.25 mg of trypsin (Sigma, T1426) per 25 mg of protein at 37 °C for 30 min. Reactions were terminated by adding 6X Laemli’s sample buffer followed by boiling at 95 °C for 5 min and immunoblotting.

### ATP measurement

ATP measurement was adapted from previously published protocol^54^. Briefly, five age-matched flies of wild type and *ringer* mutants were homogenized in 100 μl of extraction buffer (6 M guanidine-HCl, 100 mM Tris and 4 mM EDTA, pH 7.75) and 10 μl of samples were taken for measuring protein concentration using Bradford assay. The rest were transferred to liquid nitrogen immediately, followed by boiling for 5 min. Supernatants were collected after the samples were centrifuged at 14,000 rpm for 3 min at 4 °C, which were then diluted (1:750) with dilution buffer (100 mM Tris and 4 mM EDTA, pH 7.75) and mixed with luminescent solution (Enliten kit, Promega, FF2000). The luminescence was measured by a luminometer (Molecular Devices) and the relative ATP levels were calculated by dividing the luminescence by the total protein concentration. Average ± SEM is from n = 3 experiments.

### Pharmacological treatments

L-DOPA treatment was based on previously published methods^45^ with minor modifications. Briefly, freshly eclosed flies of wild type and *ringer* mutants were transferred to food containing 1 mg/ml L-DOPA (Sigma, D9628) every third day for the duration of the assays.

NAC treatment was adapted from previously published methods^27^. Freshly eclosed flies of wild type and *ringer* mutants were transferred to food containing 5 mg/ml NAC (Sigma, A9165) every third day for the duration of the assay.

Rotenone treatment was performed according to previously published methods^55^ with some modifications. Young adult flies after eclosion were transferred to standard fly food bottles containing filter paper soaked in 500 μM rotenone (Sigma, 45,656) solution dissolved in DMSO. Fresh rotenone-soaked filter paper was prepared every third day for placing on fresh food before experimental flies were transferred.

Following all of the above treatments, climbing ability using RING assay was checked as described above. MitoSOX assay to measure mitochondrial superoxide were performed after 14-days treatment, and the number of surviving flies were checked every third day for life span analysis.

### Quantification and statistical analysis

To evaluate neurodegeneration, neuronal density and number of vacuoles were analyzed. For neuronal density measurements, number of Elav-positive neuronal nuclei were counted within 100 × 100  μm^2^ area from the mushroom bodies and subesophageal ganglia of adult brains. n = 7 brains/per genotype. Number of vacuoles were analyzed from TEM images of adult brains of specified genotypes from 25 × 25  μm^2^ area from the mushroom bodies. n = 6 brains/genotype.

To assess mitochondrial morphology, GFP-labeled mitochondria of various genotypes were imaged by confocal microscopy. Mitochondrial length and elongation were determined as previously described^53^. Briefly, from confocal slices of Z-stack images compressed using maximum intensity projections, the number of mitochondria were determined by the dots of mito-GFP staining while individual mitochondrion length was measured by freehand line tool in ImageJ (NIH, USA). Mitochondrial length was a measure of the major/longest axis. Similarly, mitochondrial area was measured by freehand selection tool and surface area analyzed in ImageJ. Total of 7 brains were analyzed for each genotype.

To assess mitochondrial function, mitochondrial membrane potential was measured by TMRM dye (ThermoFisher Scientific, T668) and superoxide levels and oxidation was measured by MitoSOX Red dye (Invitrogen, M36008). Specifically, adult brains were dissected in cold PBS and incubated in 200 nM TMRM and 5 μM MitoSOX for 30 min at room temperature, respectively. Adult brains from various genotypes were imaged using identical confocal settings. Fluorescence intensity measurements for TMRM and MitoSOX were done using Image J from confocal slices of Z-stack images compressed using maximum projection functions. A total of 7 brains were analyzed for each genotype.

DA neuron quantifications were performed from defined clusters in each brain hemisphere including the anterior PAL, T1 and Sb clusters, and posterior PPM1, PPM2, PPM3, PPL1, PPL2ab and PPL2c clusters by staining with anti-Th antibodies. n = 12 brain/genotype of 1, 7, 15 and 30 day old flies.

All genotypes listed under the same quantification groups were processed and imaged under identical parameters and settings.

Statistical analyses were performed using the Graphpad PRISM software and data are presented as mean ± SEM. Statistical significance, indicated as p value, are mentioned in the respective figure legend. For comparisons between two groups, unpaired Student’s *t* test was used. For comparison of more than two groups, ANOVA with post-hoc Tukey’s multiple comparison was used. More specifically, one-way ANOVA was used when there was only one independent variable and two-way ANOVA was used when there were two independent variables. For life span analysis, survival curves of each genotype of interest were compared using the log-rank test and represented as Kaplan–Meier curves. For all quantification, the statistical significance immediately above the bars is with respect to the control genotype for that experimental group. The abbreviation ns stands for not significant when *p* ≥ 0.05.

## Supplementary Information

Supplementary Video 1.

Supplementary Information 1.

Supplementary Video 2.

## Data Availability

The data that support the findings of this study are available from the corresponding author upon reasonable request.
