# Supplementary material for: Drosophila tubulin polymerization promoting protein mutants reveal pathological correlates relevant to human Parkinson’s disease
Source: Sci Rep. 2021 Jun 30;11:13614. doi: 10.1038/s41598-021-92738-3 (PMC8245532; doi:10.1038/s41598-021-92738-3)
Supplement: Supplementary file 2 — Supplementary Information 1. [file 41598_2021_92738_MOESM2_ESM.pdf]

SUPPLEMENTAL INFORMATION

Drosophila Tubulin Polymerization Promoting Protein Mutants Reveal Pathological Correlates Relevant to Human Parkinson's Disease

Jing Xie[1,2], Shuting Chen[1,2], Jean C. Bopassa[1] and Swati Banerjee[1]*

[1]Department of Cellular and Integrative Physiology, Joe R. and Teresa Lozano Long School of Medicine, University of Texas Health Science Center San Antonio, 7703 Floyd Curl Drive, San Antonio, TX 78229, USA

[2]Xiangya School of Medicine, Central South University, Changsha, Hunan, China, 410083

*Corresponding Author

Swati Banerjee

E-mail: banerjees@uthscsa.edu

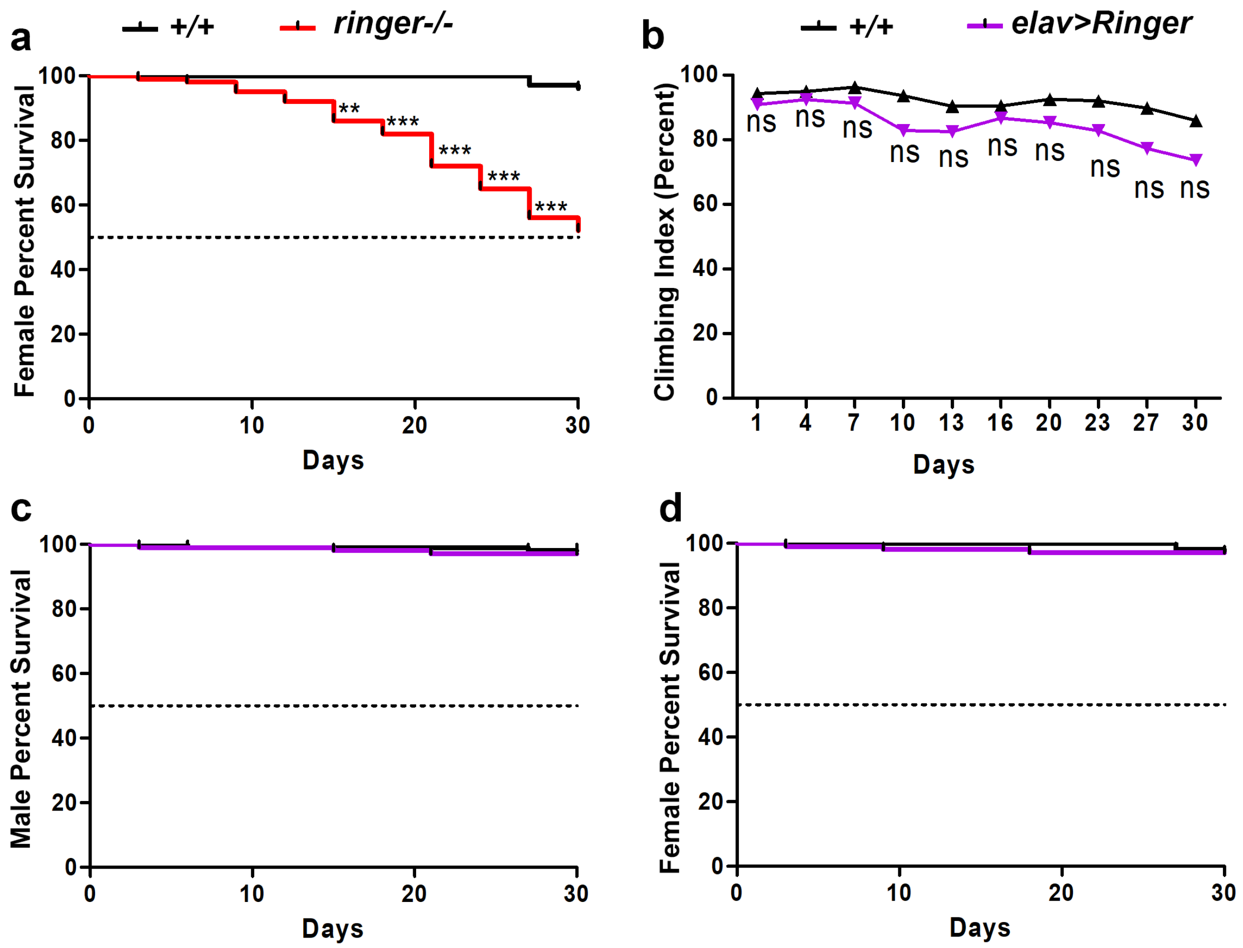

S. Fig. 1 Ringer gain-of-function in neurons do not show locomotor deficits or reduced life span. a Life span analysis of wild type (black) and *ringer-/-* (red) female adult flies. n=200 flies per genotype. Kaplan-Meyer curve, log rank test, ** $p = 0.0045$, ***$p < 0.0001$. **b** Locomotor behavior analyzed by climbing ability of wild type (black) and *elav>Ringer* (purple) male adult flies. n = 50 flies per genotype. Statistics was done using two-way ANOVA. ns $p = 0.9908$. **c, d** Life span analysis of wild type (black) and *elav>Ringer* (purple) of male **c** and female **d** flies. n=200 flies per genotype in **c, d**. Kaplan-Meyer curve, log rank test, **c** ns $p = 0.9835$. **d** ns $p = 0.6482$. (Supplement to Fig. 1)

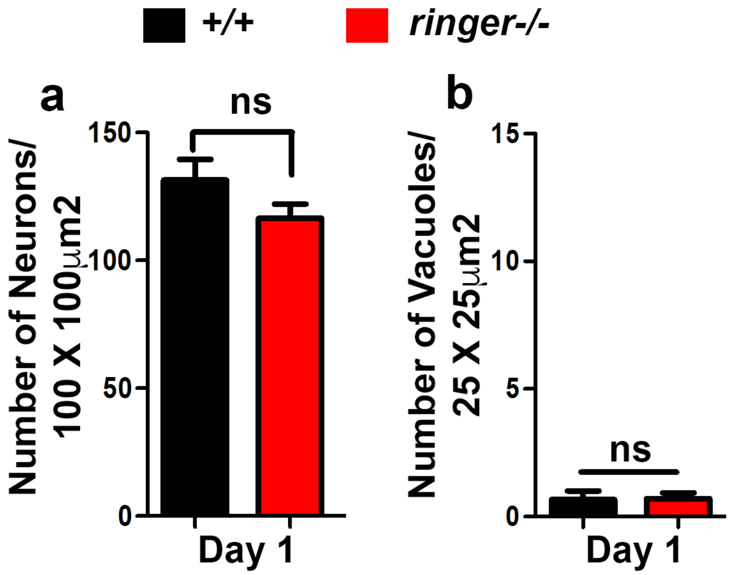

S. Fig. 2 Young day 1 *ringer* mutants do not display neurodegeneration phenotypes. a Quantification of number of neurons per 100 µm^2 of the brain from mushroom body regions in day 1 wild type (black) and *ringer-/-* (red) adults. n≥7 per genotype. **b** Quantification of number of vacuoles from 25µm^2 TEM brain sections from mushroom body regions of day 1 wild type (black) and *ringer-/-* (red) adults. n≥6 per genotype. Statistics was done using unpaired student's t-test. **a** ns p = 0.4048**. b** ns p = 0.2046.

(Supplement to Fig. 2)

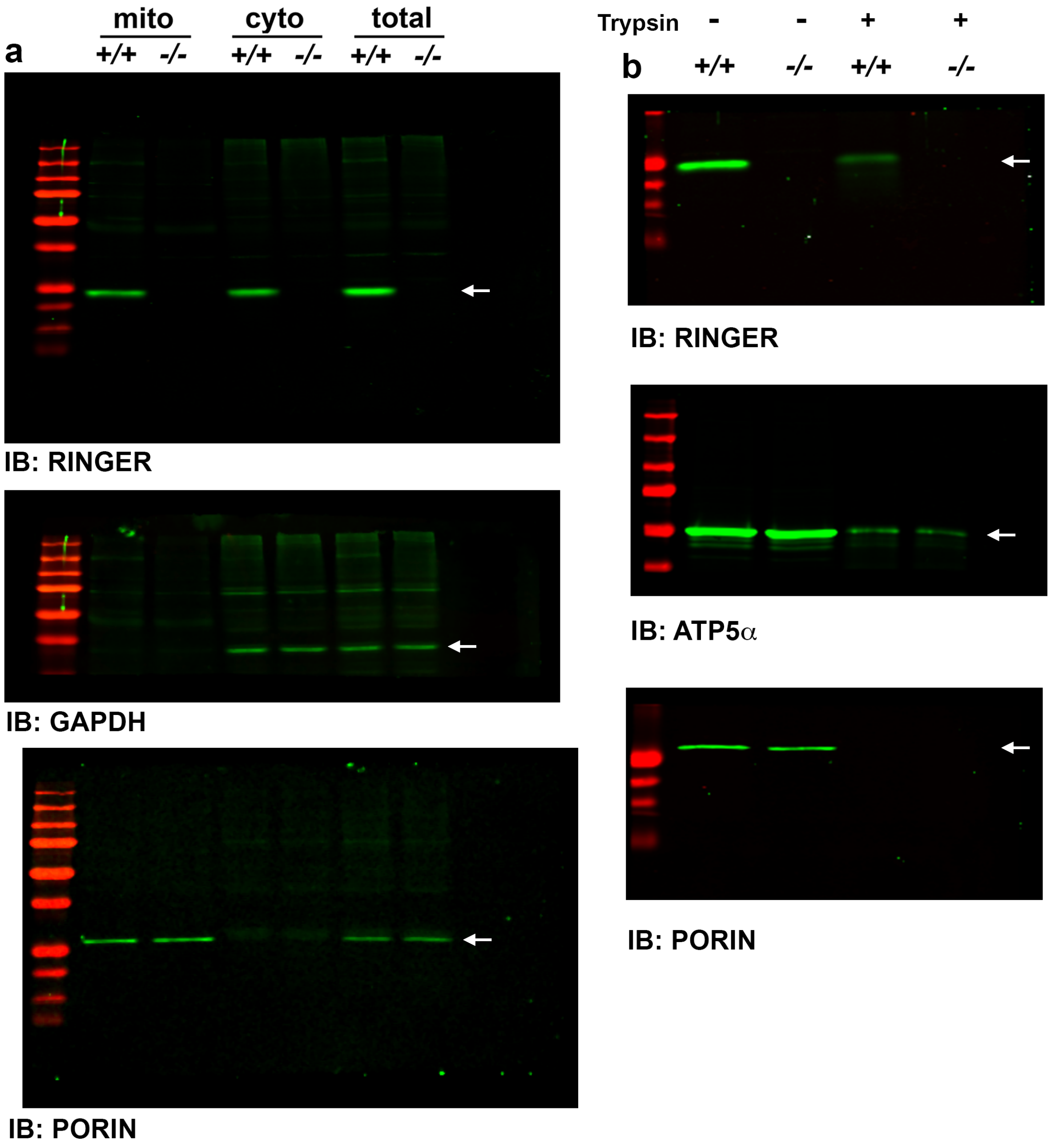

S. Fig. 3 Presence of Ringer in subcellular mitochondrial fractions of adult *Drosophila* brain. a Immunoblots, parts of which are represented in Fig. 3, of subcellular fractionation of wild type (+/+) and *ringer* mutant (-/-) adult brain probed for anti-Ringer (white arrow), anti-GAPDH (white arrow) and anti-Porin (white arrow). **b** Immunoblots, parts of which are represented in Fig. 3, of untreated (-) and trypsin

treated (+) mitochondrial fractions of wild type (+/+) and *ringer* mutant (-/-) adult brains probed for anti-Ringer (white arrow), anti-ATP5α (white arrow) and anti-Porin (white arrow). (Supplement to Fig. 3)

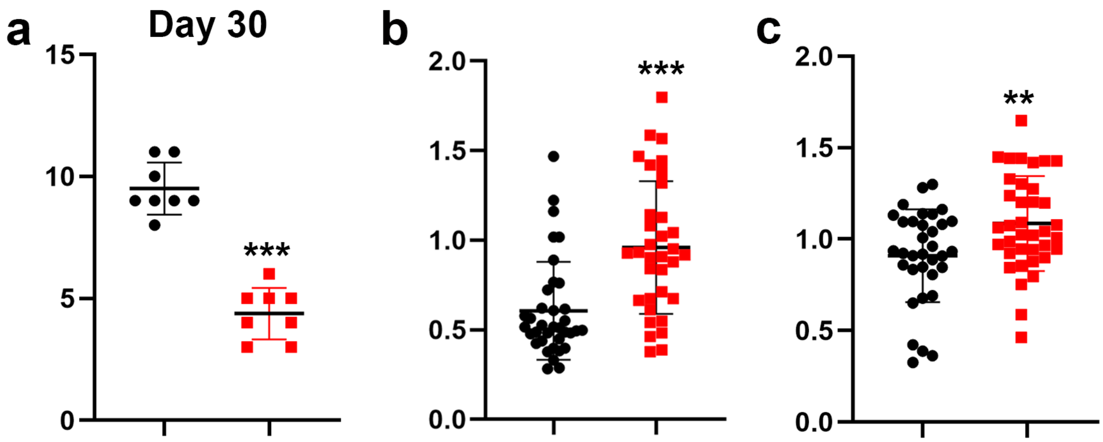

S. Fig. 4 Loss of Ringer display mitochondrial abnormalities. a-c Quantification of number of mitochondria **a**, area of mitochondria **b** and length of mitochondria **c** in day 30 flies of *elav>mito-GFP* (black) and *elav>mito-GFP,ringer-/-* (red). n ≥ 7 brains for each genotype. Statistics was done using unpaired Student's t-test. **a** ***$p < 0.0001$, **b** *$p = 0.0473$, **c** **$p = 0.0058$. (Supplement to Fig. 4)

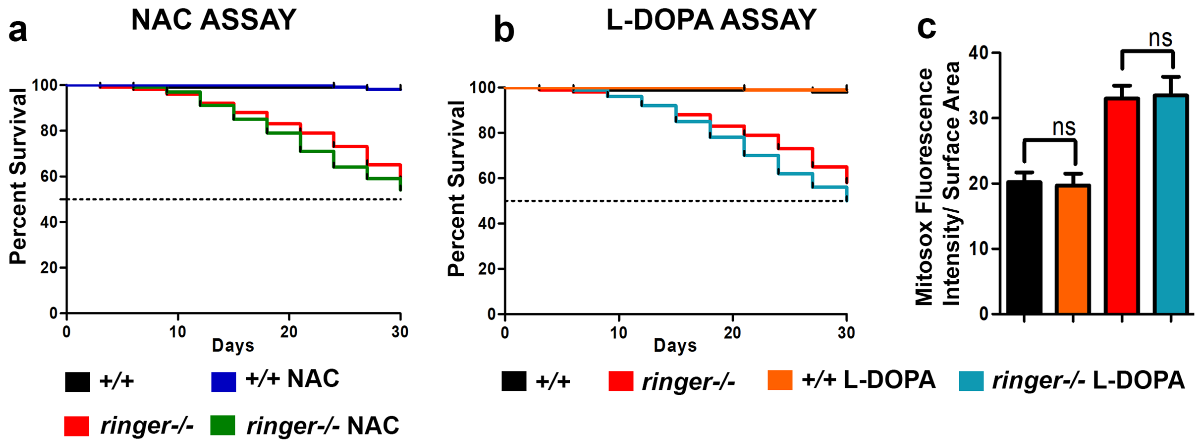

S. Fig. 5 Phenotypic consequences of NAC and L-DOPA treatment of *ringer* mutants.

a Quantification of life span analysis of wild type untreated (black), wild type treated with NAC (blue), *ringer-/-* untreated (red) and *ringer-/-* treated with NAC (green). **b, c** Quantification of life span analysis **b** and MitoSOX red fluorescence intensity **c** of wild type untreated (black), wild type treated with L-DOPA (orange), *ringer-/-* untreated (red) and *ringer-/-* treated with L-DOPA (teal). n=200 flies for life span analysis and n≥7 brains per genotype for MitoSOX assay. Log rank test was used for life span analysis. Statistics was done using unpaired student's t-test for quantification of MitoSOX fluorescence intensity. **a** +/+ with +/+ treated with NAC p = 0.9980, *ringer-/-* with *ringer-/-* treated with NAC p = 0.4673. **b** +/+ with +/+ treated with L-DOPA p = 0.1774, *ringer-/-* with *ringer-/-* treated with L-DOPA p = 0.7576. **c** +/+ with +/+ treated with L-DOPA p = 0.8397, *ringer-/-* with *ringer-/-* treated with L-DOPA p = 0.8954. (Supplement to Fig. 6)

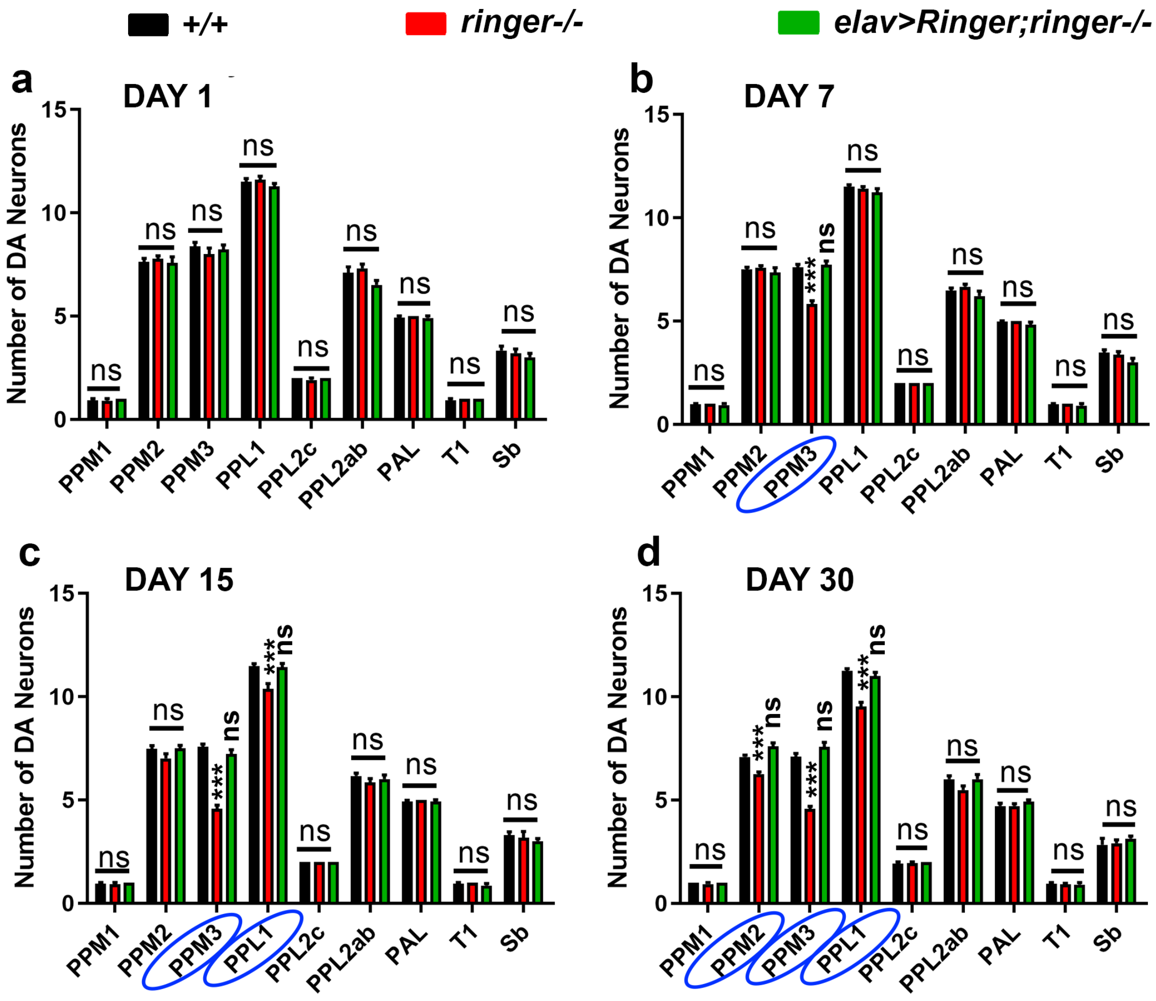

S. Fig. 6 Progressive dopaminergic neuronal loss in *ringer* mutants. a-d Quantification of DA neurons of day 1 **a**, day 7 **b**, day 15 **c** and day 30 **d** flies of wild type (black), *ringer* mutants (green) and *ringer* rescue as seen in *elav>Ringer, ringer-/-* (green). n=12 brains (~24 brain hemispheres) for each genotype. Statistics was done using one-way ANOVA and Tukey's multiple comparison. (Supplement to Fig. 7)

Day 1 :

PPM1 *ringer-/-* ns $p = 0.999$, *ringer* rescue ns $p = 0.8839$, PPM2 *ringer-/-* ns $p = 0.7458$, *ringer* rescue ns $p = 0.947$, PPM3 *ringer-/-* ns $p = 0.1675$, *ringer* rescue ns $p = 0.7427$, PPL1 *ringer-/-* ns $p = 0.8461$, *ringer* rescue ns $p = 0.424$, PPL2c *ringer-/-* ns $p = 0.8637$, *ringer* rescue ns $p > 0.9999$, PPL2ab *ringer-/-*

ns $p = 0.5494$, *ringer* rescue ns $p = 0.2785$, PAL *ringer-/-* ns $p = 0.9031$, *ringer* rescue ns $p = 0.9964$, T1 *ringer-/-* ns $p = 0.9238$, *ringer* rescue ns $p = 0.9174$, Sb *ringer-/-* ns $p = 0.8585$, *ringer* rescue ns $p = 0.3332$.

Day 7:

PPM1 *ringer-/-* ns $p = 0.966$, *ringer* rescue ns $p = 0.9689$, PPM2 *ringer-/-* ns $p = 0.8285$, *ringer* rescue ns $p = 0.6415$, PPM3 *ringer-/-* *** $p < 0.0001$, *ringer* rescue ns $p = 0.7525$, PPL1 *ringer-/-* ns $p = 0.6932$, *ringer* rescue ns $p = 0.1783$, PPL2c *ringer-/-* ns $p > 0.9999$, *ringer* rescue ns $p > 0.9999$, PPL2ab *ringer-/-* ns $p = 0.3614$, *ringer* rescue ns $p = 0.2981$, PAL *ringer-/-* ns $p = 0.9633$, *ringer* rescue ns $p = 0.6673$, T1 *ringer-/-* ns $p = 0.9653$, *ringer* rescue ns $p = 0.9409$, Sb *ringer-/-* ns $p = 0.8575$, *ringer* rescue ns $p = 0.0773$.

Day 15：

PPM1 *ringer-/-* ns $p = 0.9972$, *ringer* rescue ns $p = 0.9508$, PPM2 *ringer-/-* ns $p = 0.0793$, *ringer* rescue ns $p = 0.8762$, PPM3 *ringer-/-* *** $p < 0.0001$, *ringer* rescue ns $p = 0.0884$, PPL1 *ringer-/-* *** $p < 0.0001$, *ringer* rescue ns $p = 0.9525$, PPL2c *ringer-/-* ns $p > 0.9999$, *ringer* rescue ns $p > 0.9999$, PPL2ab *ringer-/-* ns $p = 0.2075$, *ringer* rescue ns $p = 0.7089$, PAL *ringer-/-* ns $p = 0.9091$, *ringer* rescue ns $p = 0.9999$, T1 *ringer-/-* ns $p = 0.9619$, *ringer* rescue ns $p = 0.8748$, Sb *ringer-/-* ns $p = 0.882$, *ringer* rescue ns $p = 0.4132$.

Day30：

PPM1 *ringer-/-* ns $p = 0.9624$, *ringer* rescue ns $p > 0.9999$, PPM2 *ringer-/-* *** $p < 0.0001$, *ringer* rescue ns $p = 0.1111$, PPM3 *ringer-/-* *** $p < 0.0001$, *ringer* rescue ns $p = 0.0917$, PPL1 *ringer-/-* *** $p < 0.0001$, *ringer* rescue ns $p = 0.4639$, PPL2c *ringer-/-* ns $p = 0.9892$, *ringer* rescue ns $p = 0.9566$, PPL2ab *ringer-/-* ns $p = 0.0864$, *ringer* rescue ns $p > 0.9999$, PAL *ringer-/-* ns $p > 0.9999$, *ringer* rescue ns $p =$

0.7064, T1 *ringer-/-* ns p = 0.9954, *ringer* rescue ns p = 0.9942, Sb *ringer-/-* ns p = 0.9755, *ringer* rescue ns p = 0.723.
